# Risk of Depression and Suicide in Diabetic Patients

**DOI:** 10.7759/cureus.20860

**Published:** 2022-01-01

**Authors:** Rasha Mohammed AbdElmageed, Suha Majeed Mohammed Hussein

**Affiliations:** 1 Department of Family Medicine, Primary Health Care Center (PHCC), Doha, QAT; 2 Department of Family Medicine, Qatar University Health Center, Doha, QAT

**Keywords:** suicidal ideation, diabetes, diabetic depression and suicide, suicide risk in diabetes, diabetic depression

## Abstract

Although mental disorders in diabetics are more prevalent than in the general population, an increased prevalence of depression, frequently leading to suicide, has been reported in individuals with diabetes mellitus. Therefore, the purpose of this review is to assess the risk of depression and suicide in diabetic patients. The prevalence of depression and suicide is high among diabetic individuals. Risk factors including history of depression, presence of comorbidity, younger age, lower education, low social support, presence of diabetic complications, poor glycemic control, and physical impairment, all increase the risk of depression among diabetics. On the other hand, female sex, the intensity of childhood trauma, a history of alcohol misuse, depression, lower level of education, comorbidities, higher blood glucose levels, and previous history of suicide, all increase the risk of suicide among diabetics. Additionally, a bidirectional relationship exists between depression and diabetes. For example, depression can cause diabetes due to the disease's psychological and psychosocial impact, microvascular brain lesions, higher glutamate levels, poor glycemic control, and medication adherence. On the other hand, diabetic patients develop depression due to the stress associated with disease management. This paper concluded that depression and suicide are both prevalent conditions among diabetic patients. The higher risk of depression and suicidality in diabetic patients emphasizes the critical need of integrating depression screening and treatment into primary healthcare settings to avoid fatal conditions in the future. However, more research is required in this area.

## Introduction and background

Diabetes is regarded as one of the world's largest epidemics and has been declared a public health emergency in a number of countries [1]. According to the Centers for Disease Control and Prevention (CDC), in the United States, approximately 26 million, or 8.3%, of adults and children suffer from type 1 diabetes mellitus (T1DM) or type 2 diabetes mellitus (T2DM), with the vast majority having T2DM. Globally, over 347 million individuals have diabetes, with males having a prevalence rate of 9.8% and women having a prevalence rate of 9.3% [2]. Diabetes is a mentally stressful condition because of the persistent and significant burden placed on diabetic patients regarding self-management of the disease. As a result of their disease, patients with diabetes encounter a variety of psychological difficulties, including adherence to lifestyle modifications and medical treatment, concerns about complications, the need for ongoing glycemic control monitoring, disabilities, interference with daily activities due to symptoms, and psychosocial problems at interpersonal and personal levels, all of which significantly cause and correlate with depression and, in some situations, can result in suicide.

Evidence also proposes that co-morbidity between depression and diabetes appears to be relatively prevalent [3]. Recent research estimated that diabetics had a twice-as-high prevalence of depression than the general public [4]. However, certain risk factors including poor diet, inactivity, irregular sleep patterns, and low socioeconomic status can play a significant part in activating shared physiological pathways that cause and promote depression and diabetes [5]. For example, chronic stress stimulates the sympathetic nervous system and hypothalamic-pituitary-adrenal axis, resulting in increased cortisol and adrenalin/noradrenalin production, respectively [6]. Chronically elevated cortisol levels and prolonged adrenalin/noradrenalin secretion can trigger depression (dopamine dysfunction) or diabetes (insulin resistance) or both (disruption of hippocampal neurogenesis) [6]. Moreover, depression is more likely to last longer and reoccur in diabetes patients than in non-diabetics and can even lead to suicide [7].

The global suicide rate is 16 per 100,000 people per year and results in the loss of nearly one million lives each year, or one death in every 40 seconds [8]. Suicide is frequently related to a mental health illness, particularly affective disorders (bipolar disorder and major depressive disorder). Previous research suggests that suicide rates are often lower among individuals with medical conditions, but in areas with high suicide rates, the presence of anxiety, depression, or diabetes is frequently implicated as a major risk factor. Though the relationship between depression and diabetes has been studied earlier, its link with suicide risk remains unknown, with evidence being scarce and contradictory at times [9].

There is a considerable gap in the studies regarding the comprehensive evaluation of suicidality and depression in diabetic patients. Therefore, the purpose of this study was to analyze the available evidence to assess the risk of depression and suicide among diabetic patients and to determine the risk factors that increase the risk of developing depression ultimately leading to suicide in diabetic individuals.

## Review

Relative risk and prevalence of depression in diabetic patients

For many years, the prevalence of symptoms of depression in diabetic individuals has been researched. In the late 1600s, physician Thomas Willis described patients suffering from diabetes as "...extreme melancholy...similar to fits or other depressions and breakdowns of the animal spirit..." Weyerer et al. discovered that the overall risk of psychiatric problems was substantially greater (43.1%) in patients with diabetes compared to healthy controls (26.2%) [10]. This research was supported by another study by Anderson et al., showing that the risk of depression was twice in individuals with diabetes compared to those without diabetes [9].

Additionally, researchers also reported in a community-based cohort study examining diabetes and depression that the prevalence and incidence of depressive disorders were much higher in diabetic patients than in the general population [11]. Furthermore, a population-based retrospective cohort research showed individuals with diabetes had 1.8 times more risk of depressive symptoms over a six-and-a-half-year period compared to those without diabetes [12]. Notably, Pouwer et al.'s findings showed that the existence of medical comorbidity can play a major part in the higher prevalence of depression in diabetes mellitus (DM) since depression rates in patients with DM who did not have any other comorbidity were equivalent to those in healthy controls [13]. However, these findings require replication in larger control trials, as this study included a small subgroup of patients with DM without any additional comorbidities compared to the other two groups in the research.

A study investigated the impact of obesity on diabetic individuals suffering from depression. This study found that depression was more prevalent in the overweight group compared to the normal-weight group [14]. This research described that once people become overweight, they can become more depressed as their BMI increases. On the other hand, obese individuals can have less concern about their weight, that is why they feel less nervous. Furthermore, elderly persons with adequate physical capabilities and activity levels may typically go for walks, do some exercise, and socialize with their neighbors; as a result, their moods can be rather pleasant, and they will be at decreased risk of depression. This is consistent with the findings of Narita et al., who found that physical activity helped alleviate depression in diabetic patients [15]. That is one possible theory that older people can be at decreased risk of depression, while other researchers suggest that older people with multiple diseases including DM were also more prone to suffer from depression. One explanation for this can be increased physical burden and physical discomfort associated with various diseases, or it can also be due to the increased financial cost. However, future studies comparing the prevalence of diabetes among different age groups regarding diabetes types are required. As only a few studies have shown the prevalence of diabetes according to the type.

The link between depression and diabetes

The relationship between diabetes and depression has gotten a lot of attention over the years. Studies have shown that analyzing the processes behind the diabetes-depression relationship appears to be a difficult but necessary effort. Recent research on diabetes and co-morbid depression suggests that diabetes can cause the brain's small vessel pathology comparable to that found in vascular depression [16]. Vascular depression is a kind of late-life depression in which lesions appear in specific parts of the brain impairing white matter pathways leading to the frontal cortex. As with vascular depression, white matter hypersensitivity is frequently observed in patients with diabetes and associated depression [17]. Another possibility for the connection between depression and diabetes is higher glutamate levels (a neurotransmitter linked to depression). Therefore, in a clinical trial, researchers boosted blood glucose levels in both sets of participants including patients with T1DM and a control group (individuals without diabetes), and examined brain activity using functional magnetic resonance [18]. Glutamate levels were significantly higher in T1DM patients than in the control group. This research implies that an increase in blood glucose levels can raise glutamate levels in diabetic individuals but this is not the case with non-diabetics. While researchers continue to look for molecular explanations for comorbid depression in diabetic patients, others write on the difficulties associated with diabetes management and coping. Diabetes-related stress and trouble coping may actually contribute to patients experiencing depressive symptoms. For example, diabetes management requires self-monitoring of blood sugar levels several times a day (via fingertip lancing), insulin injections and oral medications, care for lower limbs, adequate physical activity, and close monitoring of diet, all of which can be quite stressful for patients [19]. Additionally, diabetic patients with concurrent depressive symptomatology are likely to have the worst rates of poor glycemic control, medication adherence, and higher mortality compared to those with diabetes without depression [20]. Due to the complexity of diabetes management, some patients believe they lack the ability to handle diabetes; this frequently leaves them feeling hopeless and depressed [21]. However, coping with diabetes can be stressful and might cause depressive symptoms. On the other hand, depressive symptoms can result in poor self-care behaviors such as physical inactivity, unhealthy eating habits, and increased alcohol consumption, all of which are risk factors for developing diabetes [22]. As a result, other research studies have revealed a bidirectional relationship between diabetes and depression. For example, Pan et al. (2010) researched a sample of 65,381 female nurse participants and showed that both depression and diabetes are bidirectionally linked. On one hand, data suggest that depression is slightly related to the development of T2DM; on the other hand, diabetes significantly increases the risk of developing depression. Pan et al. propose two possible reasons for this finding: those who use antidepressants may suffer from more severe depression or they can have a history of depression, or antidepressants can raise the risk of acquiring diabetes. Moreover, to help explain the bidirectional relationship, Pan et al. propose that biochemical changes in the brain associated with the disease or its management place the diabetic individual at an increased risk of developing depressive symptomatology, or diabetes can cause depression as a result of the stress associated with living with the disease [23]. Other researchers have indicated similar findings; for example, researchers investigating the relationships between glucose metabolism and depressive symptoms in a sample of individuals aged 50 years and older discovered a bidirectional relationship between depressive symptoms and diabetes in individuals aged 52 to 64 years [24].

Factors that increase depression risk in diabetic patients

Risk factors for the presence of depression among type 2 diabetic individuals include the previous history of depression, presence of comorbidity, younger age, lower education, low social support, low socioeconomic status, presence of diabetic complications, poor glycemic control, and physical impairment. Similarly, some studies investigating the link between depression and diabetes showed that type 2 diabetic females are twice as likely to experience depressive symptoms as men with type 2 diabetes, a finding that is comparable with depression rates for men and women in the general population [25]. However, several studies show that certain diabetic women may experience worse health outcomes than males with diabetes. For example, researchers assessed physical impairment in men and women with and without diabetes. According to the findings, women with diabetes experience significantly higher rates of physical disability compared to diabetic men [26]. Furthermore, regardless of age, women with diabetes suffer more from impaired eyesight and mobility impairments than males with diabetes, ultimately placing them at higher risk of depression. In a study investigating the biological risk factors for depression in diabetic women and men, researchers discovered that higher rates of depressive symptoms in males were related to an increased risk of obesity and diabetes problems [27]. Rates of depressive symptoms appear to vary between men and women with diabetes, depending on biopsychosocial variables such as physical disability, obesity, and disease consequences.

Furthermore, Fisher et al. conducted longitudinal research on 508 T2DM patients tested three times over 18 months (baseline, nine months, and 18 months) [28]. Major depression, as measured by the “Composite International Diagnostic Interview” was present in 14.9% of patients at baseline and 19.8% at some stage throughout the research. This study also showed that comorbidity and younger age were independently related with maintenance of major depression across time, while female, less education, younger age, higher glycosylated hemoglobin (HBA1c) values, and higher comorbidity were independently related with the persistence of increased depressive symptoms.

Prevalence and risk of suicide in diabetes mellitus

Although the link between diabetes and depression is well-established, the same cannot be said for the link between the risk of suicide and diabetes. Numerous studies on suicide risk have reported case studies of diabetic patients who demonstrated poor self-care as an indirect predictor of self-destructive behavior and demoralization. Additionally, some researchers, for example, Silverstein et al. warned of a possible upsurge in the risk of suicide among type 1 diabetic adolescents [29]. Similarly, Kyvik et al. observed that young males with insulin-dependent DM were at more risk of suicide. This study concluded that suicide can be under-recognized as a root cause of death in these patients, but additional research studies have not been conclusive [30].

Only a few studies have reported an increase in the suicide rate among diabetic patients. For example, Tseng followed 256,036 Taiwanese diabetes patients from the years between 1990 and 2001 and discovered that 0.8% of deaths occurred as a result of suicide (0.14% of the total patients). Although this study did not provide any findings regarding types of diabetes, researchers believed that the findings strongly reflected the death among patients with T2DM [31]. Similarly, according to a meta-analysis, the suicide rate in diabetes patients is 2.35 per 10,000 person-years, implying that around 94,000 suicides occur each year among diabetes patients globally [32].

Another study assessed the suicide attempt prevalence and risk factors in type 2 diabetic individuals in a Mexican community; normal BMI, depression, and younger age were all associated with a suicide attempt [33]. According to this study, suicidal behavior was found more prevalent in type 2 diabetic individuals than in the overall population in Mexico (11.6% in diabetic patients versus 5.2% in the general population). This study's findings also indicated a high occurrence of suicidal behavior in type 2 diabetic patients. As a result, mental therapies to prevent depression and suicidal behavior are important to decrease the risk of suicide among diabetic individuals. Additional research with larger sample sizes is required to replicate and validate these findings.

Furthermore, a study revealed that approximately 13.3% of African Americans suffering from T1DM attempted suicide at some point in their lives [34]. A natural comorbidity survey also indicated that the prevalence of suicide among diabetics was also higher compared to the 4.6% of the general population who reported attempting suicide at least once in their lifetime.

Factors that increase suicide risk in diabetic patients

Numerous demographic factors have been investigated for their possible relationship with suicidality in people with DM. A study revealed that males appear to be at a higher risk of committing suicide. Another research conducted on a sample of Nigerian patients showed a lower level of education was connected with an increased risk of suicide [35]. Research data on suicide risk differs by age, sex, and diabetes type. Wibell et al. discovered that male patients were at more risk of suicide; as nine suicides occurred, whereas only 4.5 suicides were expected (95% confidence interval [CI]: 1.0-3.8, standardized mortality ratio: 2.0) [36]. When comparing diabetes types, type 1 diabetic individuals committed suicide compared to three persons with T2DM (v2 = 2.56; p = 0.11) [37]. Goldston et al. investigated the prevalence of suicidal conduct and suicidal ideation (SI) in 91 DM adolescents [38]. This study discovered that the lifetime prevalence of SI was 26.4% (n = 24 of 91) in diabetic adolescents, which seemed to be greater compared to the rates observed in the general population. This research also revealed that the length of T1DM and the prevalence of mental issues were associated with SI in adolescents. However, suicide attempts were not more prevalent in the diabetic group than in the overall population. Additionally, Quan et al. discovered that DM does not increase the risk of suicide in elderly participants [39].

In 2009, Radobuljac et al. discovered that adolescent females with DM had a significantly greater prevalence of SI than males (p = 0.001) [40]. In a clinical trial, researchers investigated self-injurious behavior in a sample of 126 patients and discovered that 38% of those who committed acts of self-harm did go through treatment modification (neglecting insulin to trigger hyperglycemia or injecting greater doses of insulin to stimulate hypoglycemia) [41]. A study previously observed a higher lifetime SI prevalence in T1DM adults and adolescents compared to other people in consideration of sex and age. They emphasized that frequent screening for depression and SI should be done to facilitate early detection and intervention in these age groups [42]. A comprehensive research examined the relationship between glycemic control and suicidality. According to this study conducted on 646 diabetic patients, suicidality was shown to be strongly linked with worse glycemic control.

Additionally, SI has been linked to depressive cognition in persons with DM. A large Korean research discovered that depression and diabetes work synergistically to increase the risk of suicidal thoughts [43]. Several researchers have also reported similar findings. Other psychological factors, such as stress, can also play a role in suicidal thoughts and attempt in people with DM. Female sex, the intensity of childhood trauma, a history of alcohol misuse, and depression were all found to be strongly and independently related to attempted suicide [44]. Additionally, individuals with diabetes often have functional disabilities including amputations and comorbidities, for example, heart diseases, and the resulting low quality of life can exacerbate depression and raise the risk of suicide.

On the other hand, a study revealed that T2DM suicide attempters had significantly higher blood glucose levels than individuals with no history of suicide. Because glucose is the major source of cellular fuel in the brain, psychological functions, for example, emotion regulation, decision-making, and self-control, highly depend on intracellular glucose availability in the brain. Another study discovered that type 2 diabetic patients had the maximum suicide rates; also, suicide risk rose as blood glucose levels raised. Chung et al. reported differences in mental health according to glucose tolerance status in a sample of 34,065 subjects and discovered that suicidal attempts, suicidal thoughts, and depressive mood for two or more consecutive weeks were all associated with elevated blood glucose levels. This is because poor glycemic control results in emotional stress, while emotional stress impedes treatment adherence, resulting in poor glycemic control, which eventually increases the risk for suicide. However, this theory should be explored in the future by including a large sample size.

The literature related to risk factors for suicidal conduct in diabetic patients is sparse. However, it has been noted that risk factors for suicide in general, as well as those associated with chronic medical diseases, likely apply in the context of DM as well. Risk factors can be inherent to the patient's features, such as coping abilities, personality profile, co-occurring psychiatric disease such as depression or alcohol use disorder, and the presence of hopelessness [45]. A patient's condition can be exacerbated by situational or either disease-related risk factors such as a lack of social support, unfavorable life events, disease worsening, and the accumulation of diabetes-related comorbidities. Access to the method of self-harm is yet another aspect that influences whether a suicide plan is carried out or not. Different protective, as well as risk factors, contribute to a patient's overall suicide risk. However, the exact mechanism through which diabetes is associated with an increased risk of suicide is not fully known, and further research is needed to understand this link.

Suicide commitment with antidiabetic medications

Löfman et al. conducted a study on a Finnish population to confirm the prevalence of T1DM and T2DM in patients suspectable of committing suicide and to analyze the suicide technique used [46]. Almost half of T1DM victims selected poison as a method of suicide, which was nearly double the rate for non-DM patients; self-poisoning was similarly more prevalent among T2DM victims compared to controls. The earliest report of high dosage of insulin use with a suicide purpose in a diabetes patient dates back to 1934 when a 50-year-old female patient used 400 units of insulin reportedly out of depression as a result of financial concerns. Subsequently, a number of studies have reported cases of suicide via self-administration of insulin where the patients received medical care and were completely recovered. Another study reported that insulin overdose was used in half of the self-poisoning cases (six of 13) among T1DM victims, but only in two patients with T2DM (13%), and none in the non-diabetic reference group preferred this method for suicide. Insulin has been widely used in suicidal attempts in type 2 diabetic patients. Additionally, half of the suicide victims with T1DM were under the effect of alcohol, which when consumed in excess can increase the hypoglycemia risk [47]. Not only insulin but its constituents like insulin lispro or glargine have also been used for self-harm attempts. A study suggested that the use of insulin as a means of suicide attempt seems to be more prevalent in people with T1DM compared to the patients with T2DM. This might be due to the fact that T1DM can only be treated with insulin and hence is more easily available to these individuals. This easier availability of insulin among depressed diabetic individuals increases the risk for suicide. On the other hand, for the treatment of individuals with T2DM, several different pharmacological medications are given, which cannot be delivered by an invasive approach.

However, studies have also reported an increase in suicide cases among type 2 diabetic patients who used oral hypoglycemic agents as a means of suicide. Avcı et al. reported five cases of people attempting suicide while receiving high dosages of metformin, an oral anti-hyperglycemic medication that promotes lactate buildup. Moreover, the research describes metformin's interactions with other medications including ethanol, which may raise the risk of or induce lactic acidosis [48]. Lactic acidosis caused by metformin can result in cardiac arrest and death. Apart from the aforementioned studies, studies on suicide attempts in individuals with diabetes have reported cases of deaths associated with excessive sugar consumption, resulting in a hyperglycemic condition. An incident of deadly self-induced hyperglycemia caused by excessive sugared tea consumption has been described in a 15-year-old diabetic child. Similarly, mortality has been reported with the use of the sugary solution by a patient with DM.

## Conclusions

Diabetes is a major risk factor for the development of depression, and diabetic individuals are at more risk of developing depression compared to the general population. Depression and diabetes have a bidirectional relationship. For example, biochemical changes in the brain associated with the disease or its management, poor glycemic control, noncompliance to medications, higher glutamate levels, and trouble coping with diabetes place diabetic individuals at an increased risk of depressive symptomatology. On the other hand, diabetes can cause depression as a result of the stress associated with living with the disease and its management. Depression and diabetes co-morbidity is associated with a worse prognosis and leads to higher medical costs than either disorder alone.

Furthermore, depression in diabetic individuals can also lead to suicide. Suicidal thoughts and behaviors are a significant clinical and public health issue in diabetic patients. While some researchers suggest that DM is related to an increased risk of suicidal thoughts and attempts, others find the opposite. However, younger age, female sex, alcohol abuse, family history of suicide, and stress increase the risk of suicide in diabetics. Additionally, access to fatal means, such as oral hypoglycemic medications and insulin, appears to facilitate suicidal attempts. Therefore, regular assessment for suicidal thoughts and treatment of psychiatric symptoms can help prevent potentially deadly results.

## References

[1] Jin H, Wu S, Vidyanti I, Di Capua P, Wu B (2015). Predicting depression among patients with diabetes using longitudinal data. A multilevel regression model. Methods Inf Med.

[2] (2016). World Health Organization. Global report on diabetes. Diabetes Care.

[3] Moulton CD, Pickup JC, Ismail K (2015). The link between depression and diabetes: the search for shared mechanisms. Lancet Diabetes Endocrinol.

[4] Han SJ, Kim HJ, Choi YJ, Lee KW, Kim DJ (2013). Increased risk of suicidal ideation in Korean adults with both diabetes and depression. Diabetes Res Clin Pract.

[5] Folb N, Lund C, Fairall LR, Timmerman V, Levitt NS, Steyn K, Bachmann MO (2015). Socioeconomic predictors and consequences of depression among primary care attenders with non-communicable diseases in the Western Cape, South Africa: cohort study within a randomised trial. BMC Public Health.

[6] Chrousos GP (2009). Stress and disorders of the stress system. Nat Rev Endocrinol.

[7] Peyrot M, Rubin RR (1999). Persistence of depressive symptoms in diabetic adults. Diabetes Care.

[8] Goldston DB, Kelley AE, Reboussin DM (1997). Suicidal ideation and behavior and noncompliance with the medical regimen among diabetic adolescents. J Am Acad Child Adolesc Psychiatry.

[9] Anderson RJ, Freedland KE, Clouse RE, Lustman PJ (2001). The prevalence of comorbid depression in adults with diabetes: a meta-analysis. Diabetes Care.

[10] Weyerer S, Hewer W, Pfeifer-Kurda M, Dilling H (1989). Psychiatric disorders and diabetes--results from a community study. J Psychosom Res.

[11] Poulsen K, Pachana NA (2012). Depression and anxiety in older and middle-aged adults with diabetes. Aust Psychol.

[12] Huang CJ, Lin CH, Lee MH, Chang KP, Chiu HC (2012). Prevalence and incidence of diagnosed depression disorders in patients with diabetes: a national population-based cohort study. Gen Hosp Psychiatry.

[13] Pouwer F, Beekman AT, Nijpels G (2003). Rates and risks for co-morbid depression in patients with type 2 diabetes mellitus: results from a community-based study. Diabetologia.

[14] Chen F, Wei G, Wang Y (2019). Risk factors for depression in elderly diabetic patients and the effect of metformin on the condition. BMC Public Health.

[15] Narita Z, Inagawa T, Stickley A, Sugawara N (2019). Physical activity for diabetes-related depression: a systematic review and meta-analysis. J Psychiatr Res.

[16] Pimontel MA, Reinlieb ME, Johnert LC, Garcon E, Sneed JR, Roose SP (2013). The external validity of MRI-defined vascular depression. Int J Geriatr Psychiatry.

[17] Kumar A, Gupta R, Thomas A, Ajilore O, Hellemann G (2009). Focal subcortical biophysical abnormalities in patients diagnosed with type 2 diabetes and depression. Arch Gen Psychiatry.

[18] Tseng CH (2015). High blood sugar causes brain changes that raise depression risk. Diabetes Care.

[19] Nguyen HT, Arcury TA, Grzywacz JG (2012). The association of mental conditions with blood glucose levels in older adults with diabetes. Aging Ment Health.

[20] Gonzalez JS, Safren SA, Cagliero E (2007). Depression, self-care, and medication adherence in type 2 diabetes: relationships across the full range of symptom severity. Diabetes Care.

[21] Kent D, Haas L, Randal D (2010). Healthy coping: issues and implications in diabetes education and care. Popul Health Manag.

[22] Cheng TL, Boey KW (2000). Coping, social support, and depressive symptoms of older adults with type II diabetes mellitus. J Aging Ment Health.

[23] Pan A, Lucas M, Sun Q (2010). Bidirectional association between depression and type 2 diabetes mellitus in women. Arch Intern Med.

[24] Katon W, Fan MU, Unützer J, Taylor J, Pincus H, Schoenbaum M (2008). Depression and diabetes: a potentially lethal combination. J Gen Intern Med.

[25] Penckofer SM, Ferrans C, Mumby P (2012). A psychoeducational intervention (SWEEP) for depressed women with diabetes. Ann Behav Med.

[26] Tsirogianni E, Kouniakis F, Baltatzi M, Lavrentiadis G, Alevizos M (2010). Biological factors associated with depression in patients with type ΙΙ diabetes mellitus. (Article in Modern Greek). Psychiatriki.

[27] Farberow NL (1980). Indirect self-destructive behavior in diabetics and Buerger’s disease patients. The Many Faces of Suicide: Indirect Self-destructive Behavior.

[28] Fisher L, Skaff MM, Mullan JT, Arean P, Glasgow R, Masharani U (2008). A longitudinal study of affective and anxiety disorders, depressive affect and diabetes distress in adults with type 2 diabetes. Diabet Med.

[29] Silverstein J, Klingensmith G, Copeland K (2005). Care of children and adolescents with type 1 diabetes: a statement of the American Diabetes Association. Diabetes Care.

[30] Kyvik KO, Stenager EN, Green A, Svendsen A (1994). Suicides in men with IDDM. Diabetes Care.

[31] Tseng CH (2004). Mortality and causes of death in a national sample of diabetic patients in Taiwan. Diabetes Care.

[32] Dahlquist G, Kallen B (2005). Mortality in childhood-onset type 1 diabetes: a population-based study. Diabetes Care.

[33] Gómez-Peralta TG, González-Castro TB, Fresan A (2018). Risk factors and prevalence of suicide attempt in patients with type 2 diabetes in the Mexican population. Int J Environ Res Public Health.

[34] Kessler RC, Borges G, Walters EE (1999). Prevalence of and risk factors for lifetime suicide attempts in the National Comorbidity Survey. Arch Gen Psychiatry.

[35] Igwe MN, Uwakwe R, Ahanotu CA, Onyeama GM, Bakare MO, Ndukuba AC (2013). Factors associated with depression and suicide among patients with diabetes mellitus and essential hypertension in a Nigerian teaching hospital. Afr Health Sci.

[36] Wibell L, Nystrom L, Ostman J (2001). Increased mortality in diabetes during the first 10 years of the disease: a population-based study (DISS) in Swedish adults 15-34 years old at diagnosis. J Intern Med.

[37] Egede LE, Nietert PJ, Zheng D (2005). Depression and all-cause and coronary heart disease mortality among adults with and without diabetes. Diabetes Care.

[38] Goldston DB, Kovacs M, Ho VY, Parrone PL, Stiffler L (1994). Suicidal ideation and suicide attempts among youth with insulin-dependent diabetes mellitus. J Am Acad Child Adolesc Psychiatry.

[39] Quan H, Arboleda-Flórez J, Fick GH, Stuart HL, Love EJ (2002). Association between physical illness and suicide among the elderly. Soc Psychiatry Psychiatr Epidemiol.

[40] Radobuljac MD, Bratina NU, Battelino T, Tomori M (2009). Lifetime prevalence of suicidal and self-injurious behaviors in a representative cohort of Slovenian adolescents with type 1 diabetes. Pediatr Diabetes.

[41] Corathers SD, Kichler J, Jones NH (2013). Improving depression screening for adolescents with type 1 diabetes. Pediatrics.

[42] Bot M, Pouwer F, de Jonge P, Tack CJ, Geelhoed-Duijvestijn PH, Snoek FJ (2013). Differential associations between depressive symptoms and glycaemic control in outpatients with diabetes. Diabet Med.

[43] Roy A, Roy M, Janal M (2010). Suicide attempts and ideation in African-American type 1 diabetic patients. Psychiatry Res.

[44] Iwasaki Y, Bartlett J, O'Neil J (2004). An examination of stress among Aboriginal women and men with diabetes in Manitoba, Canada. Ethn Health.

[45] Sarkar S, Balhara YP (2014). Diabetes mellitus and suicide. Indian J Endocrinol Metab.

[46] Löfman S, Hakko H, Mainio A, Timonen M, Räsänen P (2012). Characteristics of suicide among diabetes patients: a population based study of suicide victims in Northern Finland. J Psychosom Res.

[47] Conti C, Mennitto C, Di Francesco G, Fraticelli F, Vitacolonna E, Fulcheri M (2017). Clinical characteristics of diabetes mellitus and suicide risk. Front Psychiatry.

[48] Avcı D, Çetinkaya A, Karahan S, Oğuzhan N, Karagöz H, Başak M, Erden A (2013). Suicide commitment with metformin: our experience with five cases. Ren Fail.

